# State Care of the Insane, Epileptics, Inebriates, and Habitues of Narcotics

**Published:** 1896-05

**Authors:** F. S. White

**Affiliations:** Terrell, Texas, Late Superintendent Texas Insane Asylum, Austin, Texas


For the Texas Medical Journal.

### STATE CARE OF THE INSANE, EPILEPTICS, INEBRIATES, AND HABITUES OF NARCOTICS.

BY F. S. WHITE, M. D., TERRELL, TEXAS,
Late Superintendent Texas Insane Asylum, Austin, Texas.

[Read at the 28th Annual Meeting Texas State Medical Association, Fort Worth, April 30, 1896.]

MR. PRESIDENT:—In accordance with a resolution passed by the Terrell Medical Society, in February last, two other members and myself were appointed a committee to carry out the object of the resolution, that is, to attempt to enlist the aid of other local medical societies, and individual members of the profession, throughout the State, to have a law passed regulating the practice of medicine, and to induce the legislature to make provision for the treatment and care of all her epileptic and insane population, together with the inebriates and habitues of nacotics.

I do not now propose to discuss medical legislation, but to beg your attention for a few moments to the consideration of State care of the defective classes mentioned.

These unfortunate people, in the strict construction of the law, are wards of the State, and they should be so recognized and provided for. It is in only the rarest instances that they are responsible for their deplorable condition, which might be termed the physiological result of a pathological ancestry.

The cause of these examples of a perverted constitution dates back several generations. Environment and education, to be sure, have somewhat to do with retarding or favoring the development and manifestation of an inherited unstable organization, and determine to a considerable extent the plane the individual will occupy through life.

The care and treatment of the insane is one of the most important factors connected with the administration of any form of civilized government. In the days of Galen and Hoppocrates plans and methods of rational treatment of this class were in vogue.

The temple of Saturn in Egypt and of Asclepia in Greece, were resorted to by lunatics, and there they received treatment of a remedial nature. This condition, however, did not continue long, and during the middle ages seems to have been entirely abandoned and the inhuman and barbaric treatment substituted, which continued until about the year 1750, when the condition of the insane attracted some public attention, resulting in the incarceration of large numbers in asylums, under the control of private individuals, where the unfortunate lunatic was immured in dark and loathsome cells and dungeons, flogged and half starved, and, in many instances, put to death in the most pitiless and cruel manner.

Be it said but to the disparagement and disgrace of the boasted civilization of the nineteenth century, that this condition continues and many of these inhuman abuses survived until the year 1850. The name of Pierre, and of Wm. Tuke, a modest quaker in this country are indissolubly connected with the history of the humane treatment of the insane.* Since their time, the work to which they gave such an impetus has been gradually spreading and growing until to-day the cruel treatment of the insane is almost unknown, and is not sanctioned or authorized by any country into which the light of civilization and progress has penetrated.

There was a time when our country followed in this philanthropic work, but it is now in the lead, and in every State and territory of the Union some provision is made for the treat-

*Philippe Pinel, in 1792, with his own hands removed the chains from the insane in the Bicetre in France, and inaugurated a reform in the treatment of insanity.—EDITOR.

ment and care of our unfortunate fellow men who fall mentally crippled by the wayside.

It is an indisputable proposition that it is the duty of the government to assume the care and control of the helpless creatures —stricken by the merciless hand of disease, deserted by friends, disowned by relatives, excluded from society—

"An exile from hope and from home,
A fugitive chased by despair,"

they have no place of refuge—mindless human hulks, "dead to the love and to the joys and pains of life, oblivious of the past and unconscious of the future; stirred by no ambition, capable of no effort, and unmoved by any motive,"—like Byron's bark—

"Thrown when the war of winds is o'er,
A solemn wreck on fortune's shore;
'Mid sullen calm and silent bay,
Unseen to drop by dull decay."

From a moral standpoint, there can be no question as to the State's responsibility to care for her defective population. They are human, and are our fellow-beings, rendered helpless by disease. Many of them have, at one time, been prosperous citizens, who contributed their share to the support and maintenance of the government, and now, in their time of greatest need, the State should extend a helping hand, and if possible save them from themselves.

From a remedial standpoint, there are still stronger reasons for the State assuming control of them. Insanity is a very curable disease if treated properly and treated early.

Statistics vary some on this point, but it may be safely stated that from 75 to 80 per cent. will recover if placed under proper control and surroundings the most conducive to improvement, and be given the advantages of the very best scientific medical treatment within the first week or ten days of the mental breakdown.

On the other hand, if forced to remain in filthy jails and prisons for months, then taken to an over-crowded asylum and herded together like so many sheep, and treated *en masse*, very few will make a complete recovery.

Their minds may be so patched up that they can return to their homes (and swell the recovery list of the asylums), there to sow the seeds of insanity for future generations to reap.

Please pardon a slight diversion, but I want to step aside and say that this is the most active and prolific cause of the increase of insanity about which so many pages are so hurriedly written. Religion, politics, the great clamor and rush made by our American people for sordid gain, can in no wise compare with a half restored mind and a strong, physical body in the propagation of insanity, and in fixing upon unborn progeny an unstable, nervous organization which will go to pieces when the slightest storms of life assail it. While I am off the track, and in this connection, I want to say that I am an advocate of unsexing creative classes of the insane. I have not the time to discuss this here, but hope to do so on some other occasion.

The asylums should furnish ample room for the immediate admission and continued detention of every case unless a cure be established. Under the conditions existing in this State at present, only a small proportion of the insane can ever be admitted into the asylums, and then such a press is made for room for others that many are sent out before a cure is accomplished. They return to their homes in a weakened and disabled mental condition, and attempt again to battle with the world, and to eke out a miserable support for themselves and families—soon break down again, and probably are thrown into jail to remain for months, and until the last hope of recovery is gone, when possibly they are again admitted into the asylum to become a lifetime resident.

From an economical standpoint, the arguments are just as convincing that the State should take the care and control of the defective population. If confined in jails or poor houses or kept by some private party, the cost is paid by the county, and at last comes out of the taxes paid by the people, and amounts to more than if supported in State institutions. I some time ago ascertained this fact, by correspondence with a number of sheriffs.

What I have said with special reference to the insane, applies with equal force to the unfortunate victims of epilepsy, who, while not strictly classed with the insane, are nevertheless mentally unbalanced, and should all be under State care. They certainly constitute a most disagreeable, worthless and disturbing element of society. This peculiar affliction renders its victims unfit for citizenship. They are irritable and combative; unable to pursue any occupation continuously, they soon become a charge upon the county in which they live.

If the State were to make ample provision for all these cases where they could be sent and treated scientifically upon the very inception of the disease, many could be permanently restored. As it is, they are practically excluded from the asylums, and continue to have convulsions, and become more and more certainly incurable, until, as is too often the case, they commit some crime at which all society is horrified, when they may be admitted to some society as a last resort.

The main reason why epilepsy is considered, and is so incurable, as ordinarily encountered, is because very few cases ever receive any rational treatment until the disease is too firmly fixed to ever be eradicated.

Structural changes in the nervous system take place rapidly, under the terrible strain placed upon it by the convulsive seizures peculiar to this affection. The habit becomes a fixed condition, and science stands in helpless awe before these creatures writhing and groaning in the agony of a nervous explosion, and must acknowledge that it has nothing curative to offer.

As I have said on other occasions, I believe that the time will come when this "opprobrium medicorum" will be relieved in a very large per cent of recent cases.

The first institution for the exclusive employment and care of epileptics was established in France, about the middle of the present century, and is still in active operation. This subject has been agitated to a considerable extent recently, in this country, and has resulted in the "establishment of colonies, or the institution of necessary preliminary measures, in the States of Ohio, New York, Pennsylvania, Massachusetts, and Maryland. The colony system has been adopted in nearly all of these States.

The unfortunate inebriate and habitue of narcotic drugs is as truly the victim of disease as are the insane or epileptic.

There is a time in the life of all these cases, just as in cases of insanity and epilepsy, when the disease can be cured. With the exception, possibly, of the confirmed habitue of one or two narcotics, the slaves of poisons can, in very large numbers, be cured. Of course, like all other diseases engrafted upon an unstable nervous organization, it may return, but many can, by proper restraint and treatment, be permanently restored and returned to society, and become useful citizens instead of continuing a living burden and disgrace on relatives and friends. As a rule, this class does not possess as defective a nervous organization as does the lunatic or epileptic, and, as a consequence, treatment offers better results.

Now, gentlemen, this is a big subject, and I have left many phases of it untouched; and I hope a movement will be inaugurated here that will culminate in the State making provisions for all these unfortunate people according to the most modern plan. If you will bear with me for a few more moments, I desire to briefly describe a system which I believe to be the best and most feasible that could be inaugurated in Texas.

The ideal plan for taking care of all these cases would be to have a separate institution for each class, located in different parts of the State, but this would necessitate the expenditure of a sum of money that it would be impossible, under existing conditions, for Texas to make for some time yet, and a sum out of proportion to the benefits to accrue.

The plan that I would suggest is such as I have outlined and described several times,* and may be familiar to some of you. I advocate this plan because I believe it can be adopted in Texas, and that it will meet the requirements.

I would have but one institution, I might say, an institution under one management, but with three distinct departments—one for the insane, one for epileptics, and one for inebriates and habitues of narcotics.

There should be a superintendent in charge of the entire institution, and he should be selected, not on account of any political qualifications, but solely on account of his ability, competency, and peculiar fitness for the trust. Each one of these departments should be in charge, under the supervision and by the selection of the superintendent, of an assistant physician of superior qualification. These men should hold their positions during competency and good behavior.

This hospital should be located in one of the most fertile and healthy counties of the State, and not less than 1000 acres of land should belong to it. It should be built on what is termed the cottage system, which is now the accepted plan for all such buildings in all the older States where the experimental stage of providing for the defective population has been passed. The plan is more economical than the old system; is more beautiful and better in every respect; especially is this so in our Southern climate.

---

* See Texas Medical Journal.

By proper and efficient management this hospital (for I do call this institution a hospital and not an asylum) could, in a few years, be made very nearly self-sustaining; all kinds of industries could soon be connected with it; all kinds of vegetables and fruits could be produced and canned for use out of season.

Nearly all the work necessary to operate this establishment could be performed by the patients, and under such wholesome influences and environments many would recover their reason, who would remain permanently insane, if immured in long, regular corridors, with the floor shining like polished marble, and the walls vieing in whiteness with the driven snow. This immaculate order and cleanliness is a great thing with which to impress the average visitor and asylum committees; but how does it affect the unhappy inmate? I imagine that were I insane, it would be more pleasant, and far more conducive to recovery, to wander along babbling brooks, inhale the sweet perfume of nature's flowers, while my delusions were beguiled away by a delightful serenade from the Southern nightingale, and my whole being made to vibrate in unison with animated nature, than to be compelled to sit in a modern asylum corridor or sitting room day after day, scarcely permitted to move for fear of spoiling the polish on the floor or disarranging some piece of furniture, or, perchance, marched in regular order, and at the same hour, each day, to the same particular place in a well kept and regulated park, there to assume my habitual seat. Reforms, in this as well as in many other respects, must be made before our asylums fully accomplish the object for which they were built and are maintained.

