# Errata

**Published:** 1896-06

**Authors:** 


**Errata.**—In Dr. F. S. White's paper in last issue, on page 614, for "Pierre," read "Pinel"; on page 616, eleventh line from top, for "creative," read "certain."

